# Effects of solid oxygen fertilizers and biochars on nitrous oxide production from agricultural soils in Florida

**DOI:** 10.1038/s41598-020-78198-1

**Published:** 2020-12-10

**Authors:** Tanumoy Bera, Kanika S. Inglett, Guodong D. Liu

**Affiliations:** 1 Horticultural Sciences Department, University of Florida, 1253 Fifield Hall, 2550 Hull Road, PO Box 110690, Gainesville, FL 32611 USA; 2 Soil and Water Sciences Department, University of Florida, Gainesville, FL 32611 USA

**Keywords:** Agroecology, Environmental impact, Ecology

## Abstract

Elevated levels of nitrous oxide (N_2_O) emissions are a matter of concern in agricultural soils especially when flooding (hypoxic conditions) results from over irrigation or frequent rains. This study is the first to report the use of two solid oxygen fertilizers (SOFs, calcium peroxide and magnesium peroxide) to reduce N_2_O production in mineral and organic soils amended with N fertilizer in a short-term laboratory incubation besides two biochars. In general, organic soil had greater N_2_O production than mineral soil. Soils amended with nitrogen fertilizer exhibited increased N_2_O production, by 74 times in mineral soil and 2 times in organic soil. Both solid oxygen fertilizers in mineral soil (98–99%) and calcium peroxide in organic soil (25%) successfully reduced N_2_O production than corresponding N fertilized treatments. Additionally, a greater level of available nitrate–N (52–57 and 225 mg kg^−1^ in mineral and organic soil, respectively) was recorded with the solid oxygen fertilizers. Corn residue biochar with N fertilizer increased N_2_O production in mineral soil but decreased in organic soil, while pine bark biochar with N did not affect the N_2_O production in either soil. Depending on soil, appropriate SOFs applied were able to reduce N_2_O production and maintain greater nitrate–N levels in flooded soil. Thus, solid oxygen fertilizers can potentially be used as an effective way to reduce N_2_O emission from hypoxic soil in agricultural production systems.

## Introduction

Nitrous oxide (N_2_O) is a long-lasting (an atmospheric half life of 114 years) major greenhouse gas (GHG) with 298 times greater global warming potential than carbon dioxide (CO_2_)^1^. Recent studies have reported significant increase in atmospheric N_2_O concentration from 270 to 330 ppb^2^. Higher contribution of N_2_O is from the agriculture sector^3^ where emissions are high due to increased production and higher emissions. These are commonly observed during landuse changes, soil management, inappropriate use of nitrogenous fertilizer, livestock rearing, and handling of manure. N_2_O production in soils is primarily driven by heterotrophic denitrifying microorganisms under hypoxic and anaerobic conditions^4^. In addition to denitrification, several other processes can also contribute to N_2_O production including nitrification, chemo-denitrification, and reaction between nitrite and hydroxylamine^4^. Among the factors that affect the production of N_2_O are soil aeration, compaction, temperature, organic matter, pH, carbon (C) to nitrogen (N) ratio (C/N), available N and moisture content^4–6^. Precipitation has been shown to trigger soil N_2_O production that is episodic in nature^7,8^. Heavy rain events often cause flooding or create situations of standing water on the lowland soil surface leading to low O_2_ availability and lower redox potential. Presence of available carbon (C) and N in these conditions can stimulate N_2_O production through heterotrophic denitrification^9–13^.

In Florida, USA, sudden abnormal rains and hurricanes are common and can cause immediate waterlogging, which damages crops, especially vegetables due to stress related to low oxygen availability in soil^14^. Li et al.^15^ reported losses of approximately $77 million and $13 million in the Florida vegetable industry due to Hurricane Irene in 1999 and an excessive precipitation in 2000, respectively. Heavy rainfall can create water logging in Florida soils which is conducive to greater N_2_O emissions, thus increasing environmental hazards and decreasing N-use efficiency. The predicted changes in precipitation patterns throughout the world^16–18^ have further exacerbated this issue. In addition to soil saturation, N fertilization is also a measurable cause of N_2_O emission from agricultural soil^6^. In Florida, 224 kg ha^−1^ N is recommended for most vegetable crops^19^. Thus, these highly N fertilized soils possess a greater potential to produce and emit N_2_O in hypoxic condition through heterotrophic denitrification.

To alleviate the N_2_O production, the uses of biochar have been investigated. Biochar is a heterogeneous carbonaceous material produced by pyrolyzing agricultural residues, animal manures, wood waste, city garbage, etc. at different temperatures^20^ and has been used for environmental and soil management. The high recalcitrant aromatic C content of biochar has a mean residence time of 10^2^ to 10^7^ years^21,22^ and therefore tends to increase the soil carbon (C) stock and crop yield^23–26^. Biochar amended soils have shown to increase plant available nutrients, increased pH in acidic systems, affect water content, lower bulk density, and reduce aluminum toxicity^26,27^. Besides increasing crop growth and C sequestration, biochar was found to play a direct role in reducing N_2_O emissions. Variable effect of different kinds of biochars in soils with varying organic matter content has been reported^28–31^. Cayuela et al.^32^ reviewed 261 experimental treatments covering 30 peer-reviewed publications and proposed biotic and abiotic mechanisms behind the role of biochar influencing N_2_O emission from soil. Though the exact mechanism by which biochar reduces N_2_O production in soil is still unclear, the interactions between biochar properties, soil, and the sources and the chemical nature of N fertilizers appear to be the major controlling factors^30–33^. Another strategy to reduce N_2_O production within saturated soils is to increase the redox potential to inhibit heterotrophic denitrification. Solid oxygen fertilizers (SOFs) such as magnesium peroxide (MgO_2_), carbamide peroxide (CH_6_N_2_O_3_), and calcium peroxide (CaO_2_) have been shown to alleviate the hypoxic (low-oxygen stress) condition in flooded soils by increasing the redox potential^17,34^. Although SOFs have been reported to reduce hypoxic stress of plants, their role in reducing the anoxic condition in soil has not been investigated widely. Therefore, application of biochars or SOFs may provide an avenue to reduce heterotrophic denitrification loss of N_2_O from soil in waterlogged condition. The objective of this study was to evaluate the effects of SOFs and biochar on N_2_O production and emissions from two agricultural soils with considerably different organic C content with or without N fertilizer application.

## Results

### N_2_O production rate

**Figure 1:**
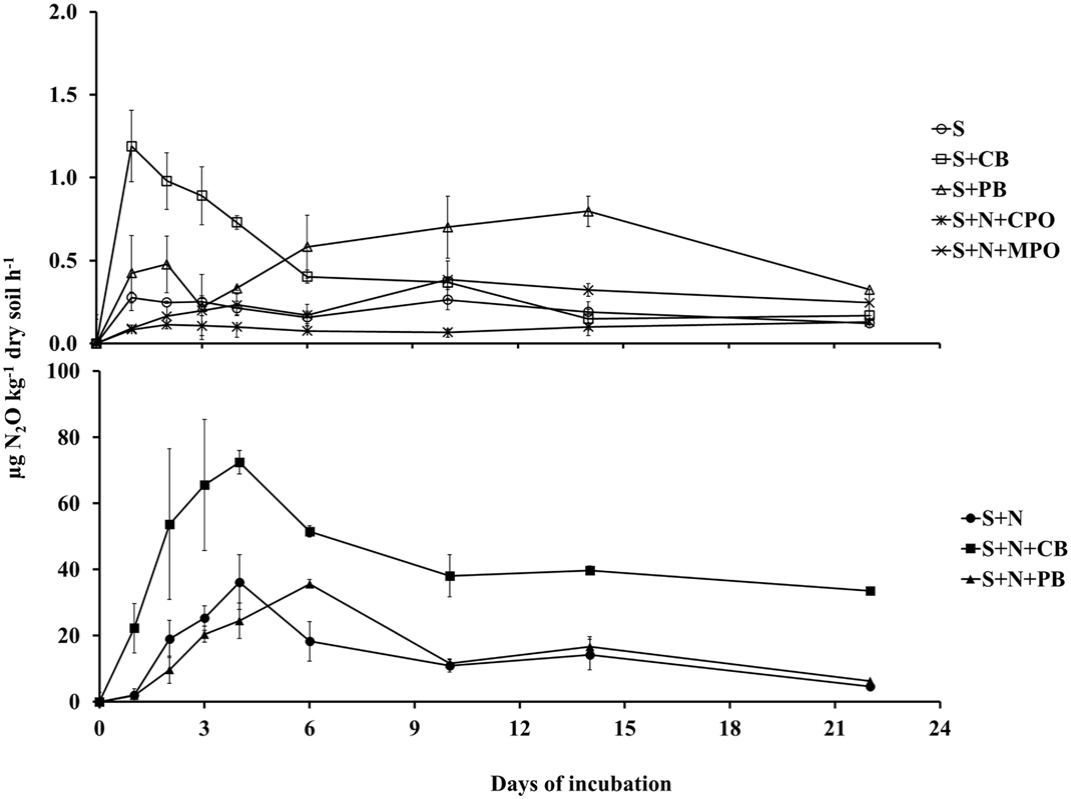
N_2_O production rate over the incubation period from mineral soil (MS). S = Control soil only; S + N = Control soil with N fertilizer; S + CB = Control soil with corn residue biochar; S + CB + N = Control soil with corn residue biochar and N fertilizer; S + PB = Control soil with pine bark biochar; S + PB + N = Control soil with pine bark biochar and N fertilizer; S + N + CPO = Control soil with N fertilizer and calcium peroxide; S + N + MPO = Control soil with N fertilizer and magnesium peroxide. Bar represents standard error, n = 3.

**Figure 2:**
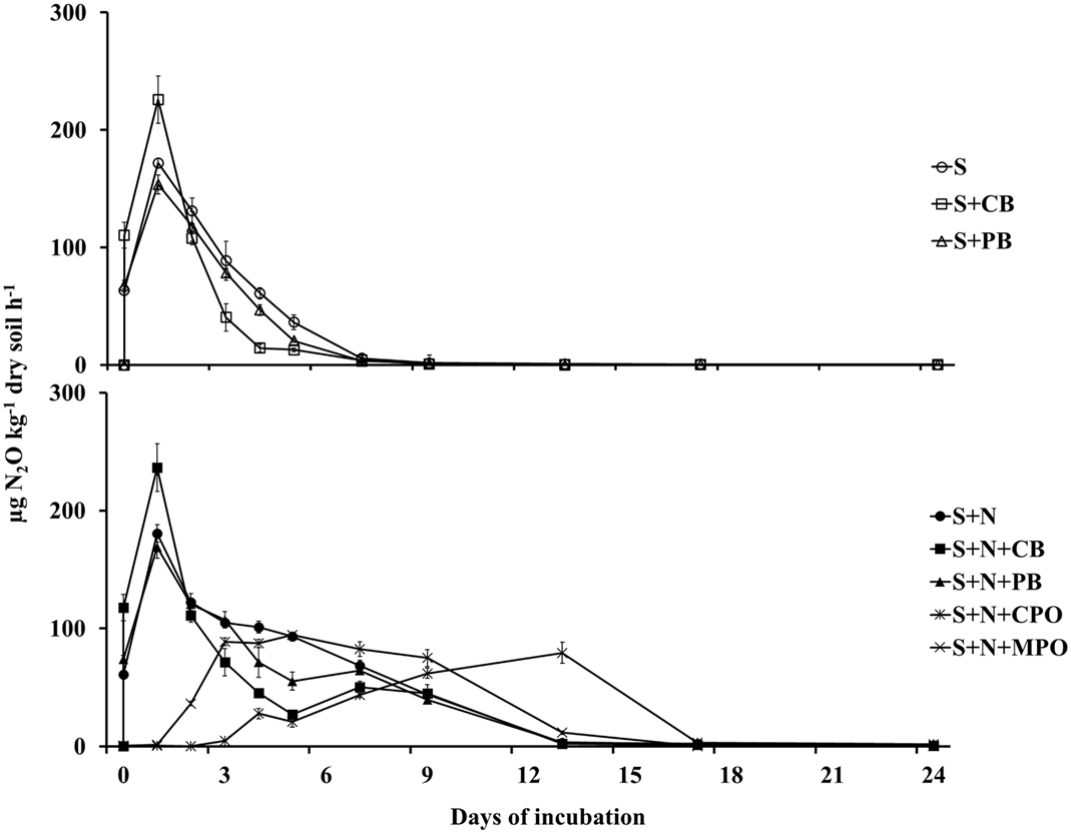
N_2_O production rate over the incubation period from organic soil (OS). S = Control soil only; S + N = Control soil with N fertilizer; S + CB = Control soil with corn residue biochar; S + CB + N = Control soil with corn residue biochar and N fertilizer; S + PB = Control soil with pine bark biochar; S + PB + N = Control soil with pine bark biochar and N fertilizer; S + N + CPO = Control soil with N fertilizer and calcium peroxide; S + N + MPO = Control soil with N fertilizer and magnesium peroxide. Bar represents standard error, n = 3.

Net nitrous oxide production (henceforth only production) rate was greater in the organic soil (OS) treatments than in the corresponding mineral soil (MS) treatments at all sampling events during incubation (Figs. 1, 2). The greatest N_2_O production rate in MS and OS soil ranged 67–73 and 181–237 µg N_2_O kg^−1^ soil h^−1^, respectively. In MS, there were two distinguishable groups among the treatments regarding N_2_O production rate (Fig. 1). All N fertilizer treatments had greater N_2_O production rates, with a peak of 73 µg N_2_O kg^−1^ soil h^−1^ with corn residue biochar and N fertilizer (S + CB + N), than the other treatments either without N fertilizer or with SOFs. Control soils without N fertilizer (S), with corn residue biochar (S + CB), and with pine bark biochar (S + PB) or control soil with N fertilizer and calcium peroxide (S + N + CPO) and magnesium peroxide (S + N + MPO) exhibited rates of N_2_O production ranging from 0.1 to 1.2 µg kg^−1^ h^−1^ N_2_O. Contrary to MS, all the treatments in OS recorded peak N_2_O production rates in the range of 80–237 µg kg^−1^ h^−1^ N_2_O. There were also differences in time to attain peak N_2_O production rate among the treatments in the two soils. In MS soil, S, S + CB and S + PB produced the greatest amount of N_2_O on the first and second day after beginning of incubation before leveling off. Interestingly, the soil with N fertilizer and CPO had similar N_2_O production throughout the incubation, while S + N + MPO recorded greatest N_2_O production rate 10 days after incubation. The control soil with N fertilizer (S + N) and S + CB + N treatments reached the peak N_2_O production rate four days after incubation. For the control soil with pine bark biochar and N fertilizer (S + N + PB), the peak rate was deferred by another two more days. All the OS treatments except two SOFs reached peak N_2_O production rate within only twenty-four hours of incubation invariably before starting diminishing. Evidently, the SOFs retarded peak N_2_O production rate attainment in OS. However, they did not reach the greater values of S + N + PB or S only, but rather retained the peak values for longer periods. The S + N + MPO had greatest N_2_O production rate after five days of incubation, which was eight days earlier than S + N + CPO’s peak N_2_O production rate.

### Cumulative N_2_O production

**Figure 3:**
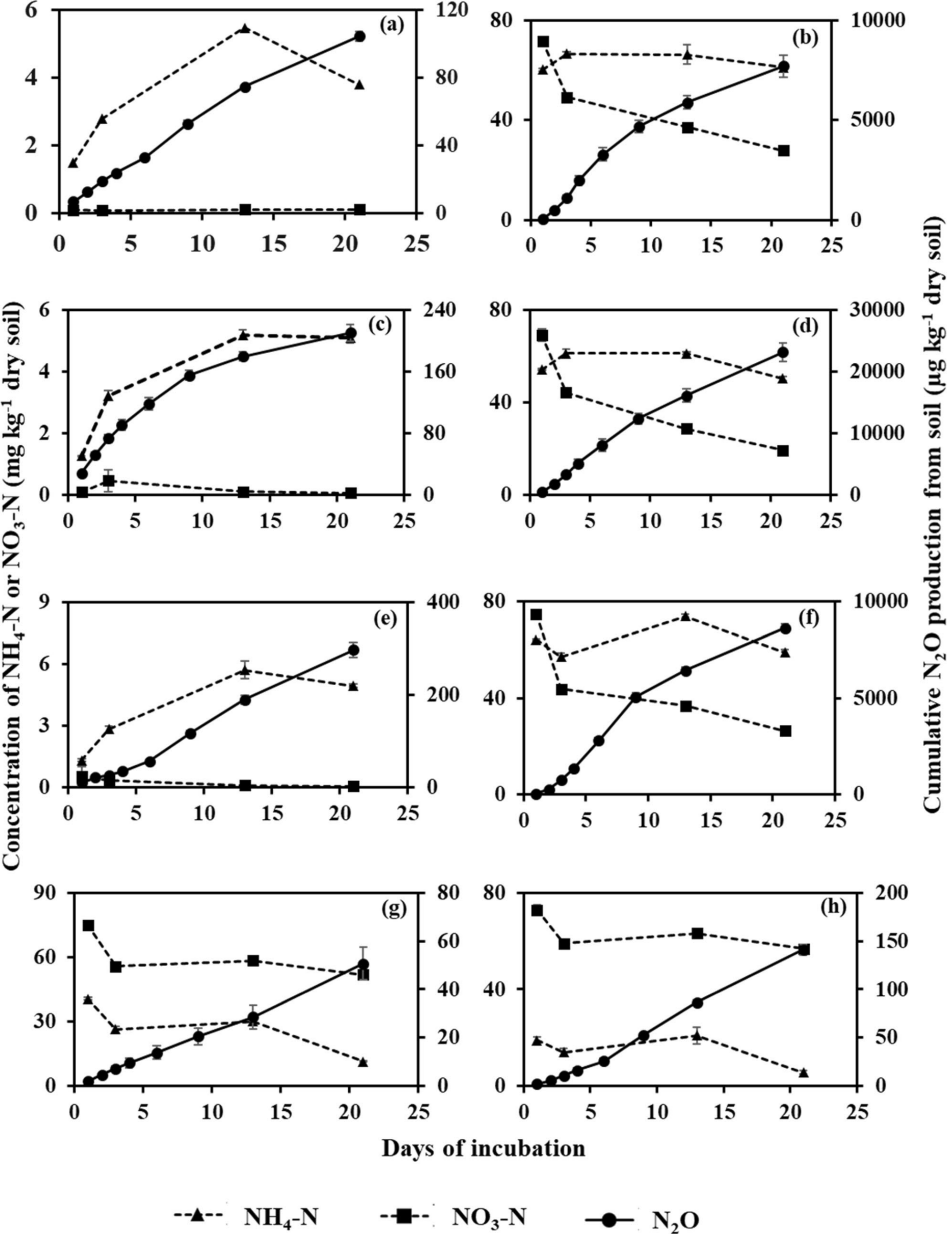
Cumulative N_2_O production, and mineral N concentration (NH_4_^+^ and NO_3_^−^) in mineral soil (MS). A = Control soil only (S); b = Control soil with N fertilizer (S + N); c = Control soil with corn residue biochar (S + CB); d = Control soil with corn residue biochar and N fertilizer (S + CB + N); e = Control soil with pine bark biochar (S + PB); f = Control soil with pine bark biochar and N fertilizer (S + PB + N); g = Control soil with N fertilizer and calcium peroxide (S + N + CPO); h = Control soil with N fertilizer and magnesium peroxide (S + N + MPO). Bar represents standard error, n = 3.

**Figure 4:**
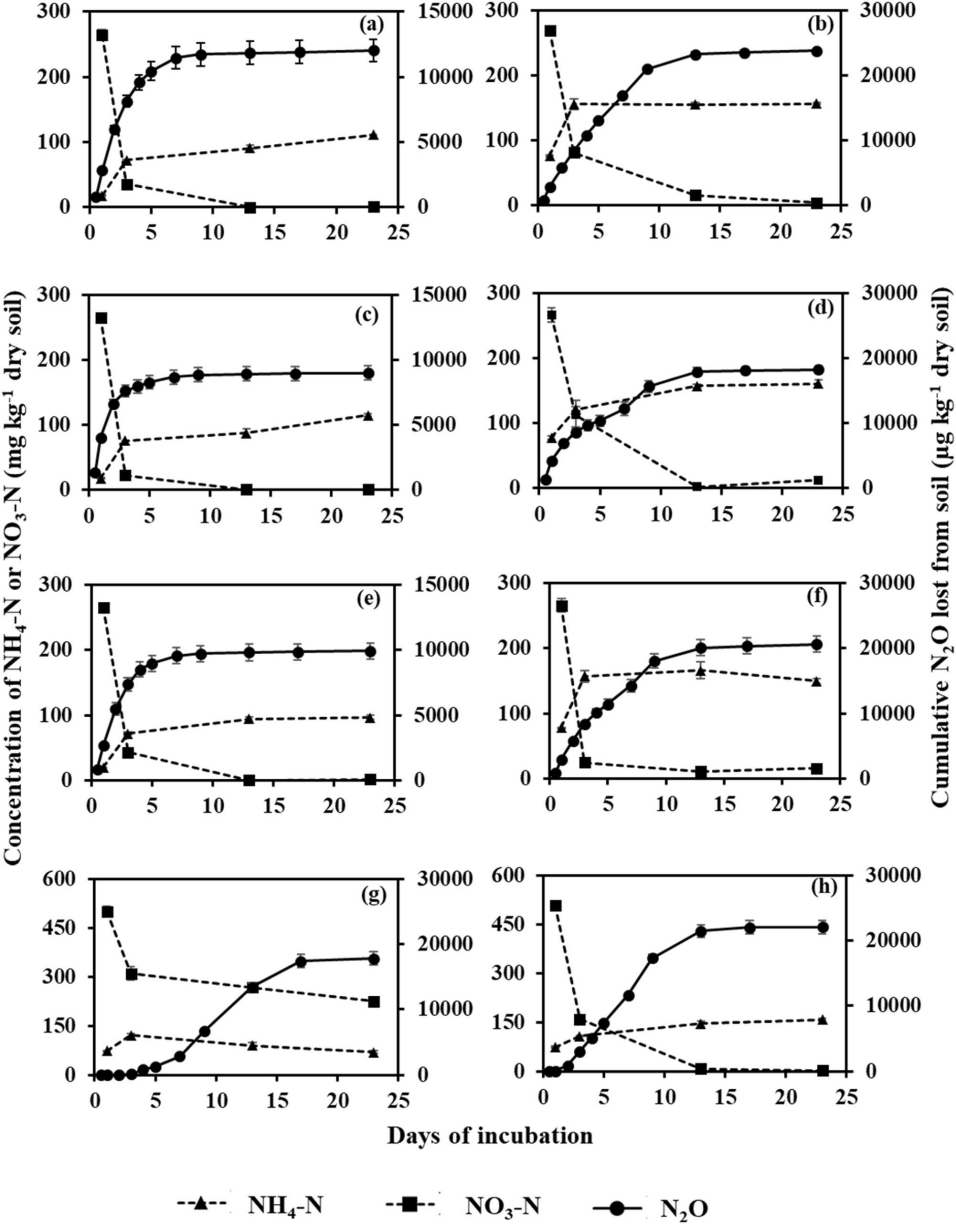
Cumulative N_2_O production, and mineral N concentration (NH_4_^+^ and NO_3_^−^) in organic soil (OS). A = Control soil only (S); b = Control soil with N fertilizer (S + N); c = Control soil with corn residue biochar (S + CB); d = Control soil with corn residue biochar and N fertilizer (S + CB + N); e = Control soil with pine bark biochar (S + PB); f = Control soil with pine bark biochar and N fertilizer (S + PB + N); g = Control soil with N fertilizer and calcium peroxide (S + N + CPO); h = Control soil with N fertilizer and magnesium peroxide (S + N + MPO). Bar represents standard error, n = 3.

For obvious reasons, cumulative N_2_O production was greater for all the treatments of OS than those of MS (Table 1; Figs. 3, 4), except the CB + N treatments. The treatments with MS can be grouped in three distinctly different groups regarding cumulative N_2_O production over twenty-one days of incubation. The group comprised of lower N_2_O production rates included S, S + CB, S + PB, S + N + CPO and S + N + MPO (Table 1). Even with N fertilizer, addition of SOFs (CPO and MPO) suppressed N_2_O production to the level of the treatments without N fertilizer. Substantively, soils with N and CPO resulted in the lowest total cumulative N_2_O production of 51 µg N_2_O kg^−1^ soil during the entire incubation period. It is also evident that presence of only biochar without N fertilizer failed to instigate N_2_O production compared to soil only. In contrast, addition of N fertilizer coupled with CB caused the greatest cumulative N_2_O production (23,185 µg kg^−1^ N_2_O) among all the treatments in MS. Pine (*Pinus* spp.) biochar along with N fertilizer application did not significantly increase total cumulative N_2_O production compared to N fertilizer-added soil. The differences among the treatments with N fertilizer in OS were not as distinct as in MS. The treatments without N fertilizer in OS, i.e. only S, S + CB, and S + CP, had cumulative N_2_O production of 12,027, 8,980, and 9,936 µg kg^−1^ soil. These values indicated significantly lower than those of the N fertilizer treatments (Table 1). In OS, the SOFs coupled with N fertilizer failed to suppress cumulative N_2_O production to the level of the treatments without N fertilizer. Rather, S + N + MPO (22,073 µg kg^−1^ soil) had similar cumulative N_2_O production to S + N (23,757 µg kg^−1^ soil). Only, calcium peroxide with N fertilizer succeeded in reducing N_2_O production significantly less than S + N, but was comparable to S + CB + N or S + PB + N.

**Table 1:** Total N_2_O production, microbial biomass C (MBC) and microbial biomass N (MBN) (mean ± standard error, n = 3) in two studied soils [organic soil (OS) and mineral soil (MS)] as influenced by biochar and soil oxygen fertilizers with or without N at the end of incubation.

	Cumulative N_2_O production (µg kg^−1^ dry soil)		MBC (mg kg^−1^ dry soil)		MBN (mg kg^−1^ dry soil)	
	MS	OS	MS	OS	MS	OS
S	104 ± 2.8^c^	12,027 ± 874^d^	87 ± 15.0^a^	1007 ± 65^a^	3.2 ± 0.50^bc^	54 ± 16.0^abc^
S + N	7705 ± 548.0^b^	23,757 ± 352^a^	88 ± 11.5^a^	850 ± 103^ab^	2.6 ± 0.27^bc^	31 ± 6.3^bc^
S + CB	210 ± 10.7^c^	8980 ± 554^d^	71 ± 5.8^ab^	552 ± 35^b^	3.8 ± 0.46^ab^	56 ± 9.7^abc^
S + CB + N	23,185 ± 1487.3^a^	18,279 ± 623^bc^	59 ± 4.8^abc^	578 ± 62^b^	5.6 ± 0.70^a^	67 ± 2.8^ab^
S + PB	29 ± 16.0^c^	9936 ± 619^d^	44 ± 5.2^bc^	635 ± 120^ab^	2.6 ± 0.37^bc^	61 ± 5.2a^b^
S + PB + N	8619 ± 189.4^b^	20,629 ± 1210^abc^	46 ± 3.8^bc^	823 ± 71^ab^	3.8 ± 0.32^ab^	83 ± 13.3^a^
S + N + CPO	51 ± 6.8^c^	17,802 ± 1019^c^	22 ± 2.4^c^	415 ± 119^b^	1.8 ± 0.11^c^	15 ± 3.6^c^
S + N + MPO	141 ± 5.5^c^	22,073 ± 1014^ab^	30 ± 7.7^c^	490 ± 78^b^	1.2 ± 0.11^c^	30 ± 7.3^bc^

S = Control soil only; S + N = Control soil with N fertilizer; S + CB = Control soil with corn residue biochar; S + CB + N = Control soil with corn residue biochar and N fertilizer; S + PB = Control soil with pine bark biochar; S + PB + N = Control soil with pine bark biochar and N fertilizer; S + N + CPO = Control soil with N fertilizer and calcium peroxide; S + N + MPO = Control soil with N fertilizer and magnesium peroxide. Values in each column followed by similar letter are not statistically different from each other by Duncan Multiple Range Test at *P* = 0.05.

**Table 2:** Initial characteristics of corn residue (CB) and pine bark (PB) biochar.

	CB	PB
pH (1:10)	8.8	8.1
Total C (%)	42.7	44.8
Total N (%)	0.9	0.2
Total P (mg kg^−1^)	39.6	61.6
**Proximate analysis (%)**		
Volatile matter	30.2	22.2
Fixed C	46.4	63.9
Ash	23.4	13.9

### Dynamics of mineral nitrogen

Figures 3 and 4 describe the pattern of mineral nitrogen (NH_4_^+^ and NO_3_^−^) in the MS and OS, respectively. In MS, the treatments were clearly segregated in producing mineral N based on the N fertilizer treatments. Soils without N fertilizer were trivial in mineral N content and range between 1.27 to 5.72 mg NH_4_^+^ and 0.06–0.54 mg NO_3_^−^ kg^−1^ soil. Soils with N fertilizer applied, except for the SOF treatments, were generally greater in NH_4_^+^ content, ranging between 50–73 mg kg^−1^, whereas soils with N fertilizer and SOFs, contained 6–41 mg NH_4_^+^ kg^−1^. For NO_3_^−^, the upper limit for soils with N fertilizer with and without SOFs were ca. 75 mg kg^−1^ soil, while the lower limit was much lower for without SOFs (19–27 mg kg^−1^) than with SOFs (52–57 mg kg^−1^). The NO_3_^−^ content steadily decreased in soil with N fertilizer and without SOFs, however, soils with N fertilizer and SOFs maintained relatively greater NO_3_^−^ levels during the incubation. In OS, irrespective of N fertilizer, soils without SOFs followed similar trends: greater concentration of NO_3_^−^ (~ 300 mg kg^−1^ soil) initially and lower concentration (< 20 mg kg^−1^ soil) at the end of the incubation. The NH_4_^+^ concentration in those treatment soils increased on the 3^rd^ day of incubation compared to the beginning and was maintained (~ 100 mg kg^−1^ soil) for the rest of the incubation period. The trends of NH_4_^+^ and NO_3_^−^ were similar for soil with N fertilizer and MPO to those soils without SOF, however, soil with N fertilizer and CPO showed different trends. The NO_3_^−^ content in S + N + CPO decreased from 501 mg kg^−1^ soil at the beginning to only 225 mg kg^−1^ soil at the end of incubation while NH_4_^+^ concentration was flattened for most of the period with a peak value of 122 on the 3rd day of incubation. It seems NO_3_^−^ content was greater than NH_4_^+^ for S + N + CPO at all sampling events while for rest of the treatment soils, NH_4_^+^ concentration became greater than the NO_3_^−^ content on the 3rd day of sampling and remained greater for the rest of the incubation.

### Soil microbial biomass C and N

Soil microbial biomass C and N in both soils were influenced by the application of biochar and SOFs with or without N fertilizer (Table 1). In mineral soils, PB with or without N fertilizer decreased MBC more than the control soil with or without N but not lower than the CB treated soils. Likewise, the soils with SOFs and N fertilizer also had lower MBC than the control soil with or without N fertilizer. The greatest MBN was recorded for MS in S + N + CB, while the lowest being the soils treated with N fertilizer and SOFs. Similar to MS, SOFs decreased MBC and MBN in the OS soil as well. Surprisingly, CB application decreased MBC in OS but not the MBN compared to the control soil without N. Thus, the impacts of biochar and SOFs, were variable considering both soils.

## Discussion

**Figure 5:**
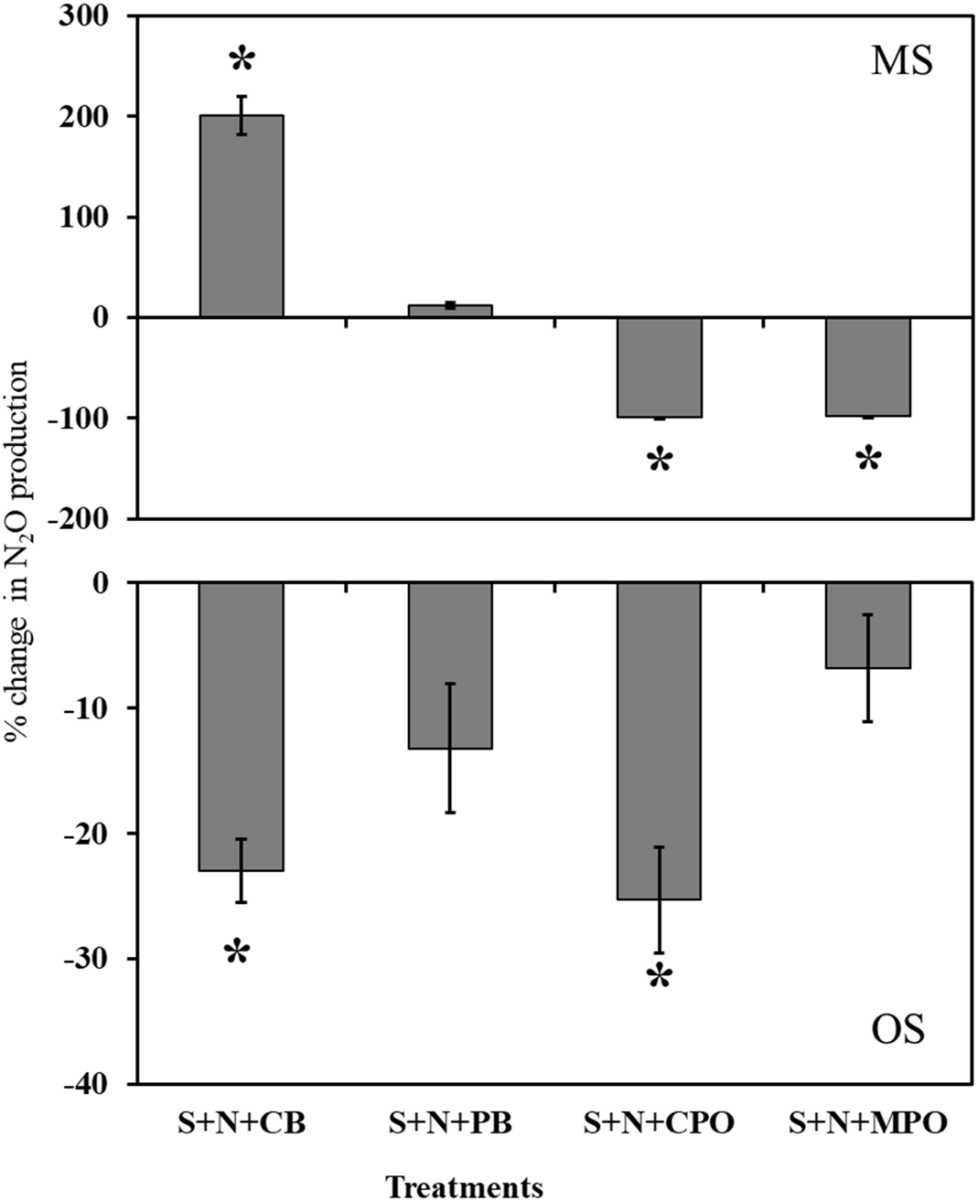
Percentage change in N_2_O production relative to soil with N fertilizer (S + N) only in mineral soil (MS) and in organic soil (OS). S + CB + N = Control soil with corn residue biochar and N fertilizer; S + PB + N = Control soil with pine bark biochar and N fertilizer; S + N + CPO = Control soil with N fertilizer and calcium peroxide; S + N + MPO = Control soil with N fertilizer and magnesium peroxide. Bar represents standard error, n = 3. * Indicates significant difference from S + N treatments in respective soil by Duncan Multiple Range Test at *P* = 0.05.

The major source of N_2_O from agricultural soil has always been attributed to applied N fertilizer^5^. In this study, N fertilizer increased 74 times and 2 times of N_2_O production in MS and OS, respectively, thereby showing the importance of soil characteristics, besides N fertilizer. Presumably, the considerable difference in organic C content (33.9% in OS and 1.2% in MS) has led to the distinct differences in N_2_O production. The major pathway of N_2_O production is heterotrophic denitrification by which NO_3_^−^ is reduced by microorganisms and used as a terminal electron acceptor^4^. In heterotrophic denitrification, microorganisms use C as energy and electron sources^35^. Thus, on average, OS produces more N_2_O given saturated moisture content than MS owing to its greater C content of OS. However, only two times increase in N_2_O in OS with N fertilizer than without N fertilizer compare to MS can be explained by following reasoning. Due to the greater C content and greater microbial biomass (Table 1) in OS, the heterotrophic NO_3_^−^ reduction process could have continued till N_2_O production was not measured in the present investigation. In extremely anoxic condition due to greater microbial O_2_ demand or prolonged water logging in soil, NO_3_^−^ is reduced by heterotrophic denitrification to N_2_^36^. In the process, N_2_O as an intermediate product of heterotrophic denitrification, is being further reduced by microbes to use as the terminal electron acceptor. The importance of organic C in N_2_O production is also evident from the attainment time of peak N_2_O production rates in both soils. The almost instantaneous peak of N_2_O production rate in OS within a day of starting the experiment may be due to readily available C and N in OS. Conversely, organic C-lacking mineral soil took almost 3 days to attain peak N_2_O production rate except soil with CB. The difference in N_2_O production due to N fertilizer is also evident from the NO_3_^−^ content of both soils with and without N fertilizer. In MS, N fertilizer increased NO_3_^−^ content from < 1 mg kg^−1^ to 71 mg kg^−1^, while for OS there was no significant difference in NO_3_^−^ content due to N fertilizer addition (Figs. 4, 5). Thus, increased availability of substrate in MS with N fertilizer led to a dramatic increase in N_2_O production despite of having low organic C content.

The effect of biochar on N_2_O production in both soils is contradictory considering the biochar and soil types (Fig. 5). In MS, CB might have provided some soluble organic C that might have acted as an energy source for soil microorganisms translating into such a greater N_2_O production with N fertilizer compared to soil with N fertilizer only. In the literature, volatile matter in biochar has been previously reported as a source of C for microorganisms^37,38^. Ameloot et al. showed a strong positive correlation between the easily mineralizable C pool and the volatile matter content of swine manure fermentation digestate and willow wood biochar with two different pyrolysis temperature (350 and 700 °C). The PB biochar effects can be similarly explained; however, the greater N_2_O production in soil with N fertilizer only indicates the importance of supply of NO_3_^−^ that is the substrate of heterotrophic denitrification^5^. The supply of NO_3_^−^ came from the applied N fertilizer in this present experiment as evidenced by the greater NO_3_^−^ content in N fertilized MS soil (Fig. 4). Thus, for the greater N_2_O production from soil both the substrate, i.e. NO_3_^−^ coming either from organic (soil amendments) or mineral (N fertilizers) sources and the source of energy for microorganism, that is C, is apparent^4^. However, the increased availability of organic C did not translate into greater soil microbial biomass for S + N + CB in MS, possibly indicating water logging stress. Given waterlogging with anoxic conditions, microbes showed greater respiration by increasing hydrogenase enzyme activity and metabolic quotient^39,40^. In this situation, microorganisms, which are facultative in nature, try to adjust with the new stressful environment by increasing respiration and losing C to the atmosphere rather than assimilating as biomass^40^. Thus, to continue profuse respiration, microbes use NO_3_^−^ as a terminal electron acceptor in absence of molecular O_2_ in a waterlogged environment^4^. This fact is also supported by steep decreases in the NO_3_^−^ concentration in N fertilized mineral soil without SOF and all the OS treatments in the present experiment. Contrarily, the profound effect of biochar is not that clear in OS. The OS is already well supplied with available organic C to microbes that the miniscule amount added through biochar is of less importance. Rather, ample availability of substrate for heterotrophic denitrification, i.e. NO_3_^−^ added through N fertilizer, resulted in greater N_2_O production than soils without N fertilizer. However, in sharp contrast to mineral soil, CB significantly reducing N_2_O production in OS indicates the controlling mechanism is a result of unclear interaction between soil and biochar. Previously, many investigators have also reported transient effects of biochars on soil N_2_O production depending on soil and biochar properties^29–32^. Biochar ash content has been the other determining factor to regulate its impact on soil N_2_O emission though there is no general agreement on the underlying mechanism among the researchers. The conflicting effects of biochar volatile matter and ash contents on GHG emissions were multiplied by biochar-soil texture interaction that has produced widely varying effects on GHG emissions^33^. So, the present experiment including CB and PB with two distinct ash and volatile content results differently in two soil differing in C and texture. In both soils, biochar seems inconsequential in regulating the dynamics of mineral N as the concentrations of NH_4_^+^ and NO_3_^−^ are similar with or without biochar in N fertilized soil. In the literature, contrasting effects of biochar on NO_3_^−^ and NH_4_^+^ have been reported depending on biochar and soil properties in flooded or field capacity moisture conditions^41,42^.

The impact of SOFs in reducing soil N_2_O production is not studied widely yet. The underlying mechanism of increasing bioavailable O_2_ under water stagnant conditions or in any aqueous solution is assumed to be based chemical equilibrium Eq. (1):

1 $${\text{MgO}}_{{2}} /{\text{CaO}}_{{2}} \left( {\text{s}} \right) \, + {\text{ 2H}}_{{2}} {\text{O }}\left( {\text{l}} \right) \to {\text{ Mg}}\left( {{\text{OH}}} \right)_{{2}} /{\text{Ca}}\left( {{\text{OH}}} \right)_{{2}} \left( {\text{s}} \right) \, + {\text{ H}}_{{2}} {\text{O}}_{{2}} \left( {\text{l}} \right) \, + \Delta {\text{H}}$$

The hydrogen peroxide from the above equilibrium, being unstable in normal temperatures, further decomposes to water and nascent oxygen. The nascent oxygen quickly forms O_2_ and becomes bioavailable to microbes or plant roots to take up for respiration as Eq. (2).

2 $${\text{2H}}_{{2}} {\text{O}}_{{2}} \left( {\text{l}} \right) \to {\text{ 2H}}_{{2}} {\text{O}}\left( {\text{l}} \right) \, + {\text{ O}}_{{2}} \left( {\text{g}} \right)$$

The decomposition of SOFs is determined by chemical equilibrium principles that in turn depend on the solubility of SOFs in water. In mineral soil, both SOFs significantly reduced N_2_O production, indicating that the amount applied was enough to provide ample O_2_ in waterlogged conditions to fulfill the requirements of microbial respiration. However, the inability of MPO to reduce N_2_O production in OS soil could be explained by the lower water solubility of MPO compared to CPO. The CPO (1.65 g L^−1^) has 19 times greater water solubility than MPO (86 mg L^−1^) at 20 °C. Organic soil, being greater in organic C, inhabiting greater soil microbial biomass requires greater amount of oxygen than the mineral soil. Less soluble MPO failed to provide the required O_2_ resulting in a similar N_2_O production as soil with N fertilizer only. Similarly, both the SOFs in MS and CPO in OS maintaining greater NO_3_^−^ level throughout the incubation period suggests that both SOFs were suitable for maintaining a greater oxygenated environment in MS. In OS, only CPO could maintain the same owing to its greater solubility, despite the greater O_2_ demand of OS microbes. Additionally, greater level of NO_3_^−^ would pose a leaching problem if there is not any plant to uptake or soil is sandy in nature. However, NO_3_^−^ leaching due to SOF is beyond the scope of current investigation to discuss.

The only point of concern is the reduction of microbial biomass in both MS and OS soils with N fertilizer and SOFs. The drastic decrease in microbial biomass in SOF treatments could also be from the possible heat and increased pH^43^. Dissociation of SOFs is an exothermic reaction resulting in increased soil pH of the medium due to formation of hydroxide^43^. At the end of experiment, mineral soil pH measured between 9.80 and 10.00 for CPO and MPO would validate the reasoning. For organic soil moderate pH change (8.25–8.35) would indicate the greater buffering capacity of organic soil than mineral soil thus having lesser impact on microbial biomass. However, the application of SOFs has been successful in alleviating hypoxic stress in corn and Italian basil^14,34^ even though it may increase the pH. Besides, in the present study, SOFs maintained greater levels of available NO_3_^−^ in flooded conditions supplying ready availability of N to plant roots. Apparently, the immediate harmful effects of SOFs on plant production is negligible if there is any.

## Conclusions

Nitrous oxide emission is a serious concern for human health, agriculture, and the environment worldwide. Findings from our study indicate that N fertilization of mineral soils is potentially more prone to increased N_2_O production. This is contrary to the general norm that organic soil tend to have higher N_2_O production than mineral soil. Both solid oxygen fertilizers in mineral soil and calcium peroxide in organic soil were successful in reducing N_2_O production while maintaining greater levels of NO_3_^−^. The corn residue biochar with N fertilizer increased N_2_O production in mineral soils but decreased production in organic soil. Pine bark biochar with N did not escalate in either of the soils indicating the mixed response of biochar on N_2_O production depending on biochar and soil type. Thus, depending on the soil, different types of solid oxygen fertilizers may be applied to alleviate hypoxic stress, decrease N_2_O production, and maintain plant available NO_3_^−^ in soil in hypoxic conditions. These findings provide a basis for future long-term laboratory incubation or field studies with different soil types and solid oxygen fertilizer rates to formulate an effective solid oxygen fertilizer technology for adaptation by growers.

## Materials and methods

### Soils, biochars and solid oxygen fertilizers

Two soils differing in organic matter content and texture were used for this laboratory experiment. Mineral soil with 1.2% organic C, Kanapaha fine sand, (loamy, siliceous, semi-active hyperthermic Grossarenic Paleaquult)^44^ was collected from UF/IFAS Plant Science Research and Education Unit, Citra (29° 24′ N 82° 08′ W). Organic soil with 33.9% organic C (Pahokee Muck, hyperthermic Lithic Haplosaprists)^45^ was collected from UF/IFAS Everglades Research and Education Center, Belle Glade (26° 39′ N 80° 37′ W). Ultisols (~ 2.8 million ha) and Histosols (~ 1.6 million ha) are major soil groups that support Florida’s agriculture (valued $104 billion yearly), including vegetable and sugarcane production^46^. The soils were brought to the laboratory in the Horticultural Sciences Department at University of Florida (UF), Gainesville, Florida, USA. The sampled bulk soil was homogenized, passed through a 2-mm sieve, and stored in polyethylene containers at − 4 °C until start of the incubation experiments. Initial physico-chemical properties of both soils are presented in Table 3.

**Table 3:** Initial physico-chemical properties of mineral (MS) and organic (OS) soil.

	MS	OS
pH (1:2)	6.3	6.9
Texture	Sand	Sandy clay loam
Sand (%)	98.8	56.0
Silt (%)	0.8	18.4
Clay (%)	0.4	25.6
Maximum water holding capacity (%)	22	128
Total C (%)	1.2	33.9
Total N (%)	0.72	3.6
Mehlich-1 P (kg ha^−1^)	50	75
Mehlich-1 K (kg ha^−1^)	20	894
Mehlich-1 Ca (kg ha^−1^)	529	9403
Mehlich-1 Mg (kg ha^−1^)	42	1560
CEC (meq 100 g^−1^ soil)	2.2	31.8

Two biochars were produced from two different feedstocks viz. corn residues and pine bark through a slow pyrolysis process in a cast iron kiln in an electric furnace at the Agriculture and Biological Engineering Department, UF, Gainesville, FL. Both residue feedstocks were chopped into small pieces (~ 2 cm in length) and sun dried to a constant moisture content before pyrolysis. Feedstocks were pyrolyzed slowly with temperature increasing at a rate of 10 °C min^−1^ until 550 °C and kept at that temperature for 4 h. Subsequently, the pyrolyzing reactor was left to cool in natural conditions overnight. Two biochars (CB-corn biochar, PB-pine bark biochar) were then oven-dried at 65 °C, crushed, sieved (2-mm), and stored in polyethylene containers at room temperature until further use. The initial properties of biochar are presented in Table 2.

The SOFs used in this experiment were calcium peroxide (CaO_2_, CPO) and magnesium peroxide (MgO_2_, MPO), provided by Solvary Interox, Inc. (Houston, TX, USA). Calcium peroxide is a pale yellow, odorless powder with a solubility of 1.65 g L^−1^ at 20 °C and pH of 11.7 of 1% aqueous suspension. Magnesium peroxide is a white odorless powder with solubility of 86 mg L^−1^ and pH of 10.3 of 1% aqueous suspension.

### Laboratory incubation experiments

Two laboratory incubation experiments were conducted separately to evaluate the effects of SOFs and biochars on N_2_O production (Experiment 1) and mineral N (NH_4_^+^ and NO_3_^−^) contents (Experiment 2) in these two studied soils. Conducting experiment 2 separately allowed us to sample soil destructively for mineral N determination without affecting N_2_O production. Both experiments were conducted simultaneously at constant temperature (23–25 °C) during March–April 2017. The treatments for experiments were: Control soil without nitrogen (S) and with 224 kg N ha^−1^ through ammonium nitrate (33.5% N) (S + N); soil with CB without N (S + CB) and with N (S + CB + N); soil with PB without N (S + PB) and with N (S + PB + N); soil with N with either CPO (S + CPO + N) or MPO (S + MPO + N). In total, there were 48 incubation units, including two soils × eight treatments replicated three times. The SOFs and biochar were applied at the rate of 0.5% (w/w). For experiment 1, 100 g of MS and 50 g of OS soil were mixed with respective treatments and incubated for ca. three weeks in loosely covered, wide-mouth mason jars (473 cm^3^) in darkness on a laboratory bench top. For experiment 2, four incubation units consisting of 75 g of MS or 37.5 g of OS were maintained separately to have 300 g of MS and 150 g of OS in total for a single replication of an individual treatment. Before starting incubation, soil and biochar for respective treatments were mixed to a uniform blend and moistened with distilled water to 60% of the maximum water holding capacity (MWHC). At this stage, units were incubated for three days to eliminate the disturbances due to sampling, processing, and storing. At the start of the experiment a nitrogen fertilizer solution made from ammonium nitrate (33.5% N) and extra distilled water was added to make up moisture content to 100% of MWHC of two soils. During the experiment, incubation units were periodically checked for evaporative water loss and distilled water was added through micropipette in an event of any moisture loss detected. For experiment 1, N_2_O was measured at pre-determined intervals that were more frequent in OS (0.5, 1, 2, 3, 4, 5, 7, 9, 13, 17 and 23 days of incubation) than MS (1, 2, 3, 4, 6, 9, 13 and 21 days of incubation). For each soil sampling event (1, 3, 13 and 21 or 23 days of incubation for MS or OS, respectively), 25 g of moist soil were destructively sampled from each incubation unit for mineral N analysis in Experiment 2. At the end of incubation, last sampling soils were used to determine soil microbial biomass C and N in respective treatments for both soils.

### Soil, biochar, N_2_O and microbial biomass analysis

Soil pH was measured in a 1:2 soil to distilled water ratio. Soil total C and N content were analyzed by dry combustion in a Thermo Flash EA 1112 elemental analyzer (CE Elantech Inc., Lakewood, NJ, USA)^22^. Extractable phosphorus, potassium, calcium, and manganese were extracted with Mehlich-1 extractant followed by determination in inductively coupled plasma spectrophotometry (ICP-AES). Texture, maximum water holding capacity, and cation exchange capacity of soil were measured following standard procedure. Mineral N (NH_4_^+^ and NO_3_^−^) of soils was extracted with 2 mol L^−1^ KCl and measured using a discreate flow autoanalyzer AQ2 (SEAL Analytical Inc. Mequon, Wisconsin). Biochar pH, total C and N were measured similar to soil with exception of the biochar ratio: water (1:10) ratio. Total P was measured after acid digestion followed by ICP-AES determination. Proximate analysis of biochar was conducted following the procedure of Bera et al.^47^. Nitrous oxide release was measured using gas chromatograph (GC-2014, Shimadzu USA) equipped with electron capture detector to measure N_2_O equipped with electron capture detector. At each sampling, a jar containing the sample was tightly capped for 12 h. After that, a 1-mL syringe was used to draw the air from the jar, and the sample was directly injected in the GC. Soil microbial biomass C and N were measured following standard procedure of chloroform fumigation and 0.5 M potassium sulfate extraction^48^.

### Data calculation and Statistical analysis

At each sampling, N_2_O concentration was calculated using the universal gas law and then dividing the concentration by 12 h to get the rate^49^. Cumulative N_2_O concentrations were estimated by linear interpolation of the hourly N_2_O concentration rate at each sampling event for each treatment in each soil. Both experiments were conducted as completely randomized design. To determine the treatment effects, data were analyzed using one-way analysis of variance in PROC ANOVA (SAS, 2015, SAS Institute Inc., Cary, NC, USA)^50^. The multiple mean separation with corresponding letter grouping method was performed using Duncan’s Multiple Range Test (DMRT) at a significance level of 5% in SAS.

## Data Availability

The datasets generated during and/or analyzed during the current study are available from the corresponding author on reasonable request.
